# Supplementary material for: Improving Sonication Efficiency in Transcranial MR-Guided Focused Ultrasound Treatment: A Patient-Data Simulation Study
Source: Bioengineering (Basel). 2023 Dec 26;11(1):27. doi: 10.3390/bioengineering11010027 (PMC10813010; doi:10.3390/bioengineering11010027)
Supplement: Supplementary file 1 [file bioengineering-11-00027-s001.zip › bioengineering-2744950-supplementary.pdf]

Supplementary Materials

Improving Sonication Efficiency in Transcranial MR-guided Focused Ultrasound Treatment: A Patient-Data Simulation Study

Changsoo Kim[1], Matthew Eames[2,3] and Dong-Guk Paeng [2,4,*]

1 Research Institute for Basic Sciences, Jeju National University; yustchangjnu@jejunu.ac.kr
2 Focused Ultrasound Foundation, matt.d.c.eames@gmail.com
3 Department of Radiology, University of Virginia, meames@fusfoundation.org
4 Ocean System Engineering, Jeju National University; paeng@jejunu.ac.kr

*Corresponding Author E-mail: paeng@jejunu.ac.kr

Table of Contents

Figure S1. Ray tracing result in KranionM.

Figure. S2. Normal vector based on the collision point.

Figure S3. An illustration of total transmission coefficient calculation strategy.

Figure S4. The implementation of transducer tilting in KranionM.

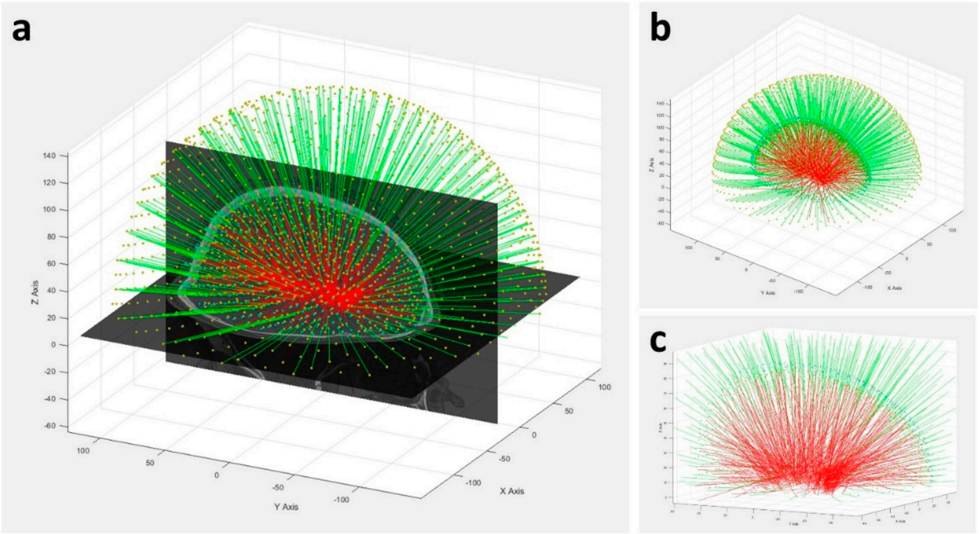

Figure S1. Ray tracing result in KranionM. The ray-tracing (two-thin-layer model) was implemented in KranionM. The transducer coordinate was drawn using gold-color dots, and the ray from the transducer to the first collision point (outer skull table) was marked using green lines. The second ray, which starts from the second collision point (inner skull table) was indicated using red lines. a) Two orthogonal CT slice images were overlapped with the transducer coordinates, rays, and collision points. b) the ray-tracing result without CT slice images. c) The zoomed-in view of (b).

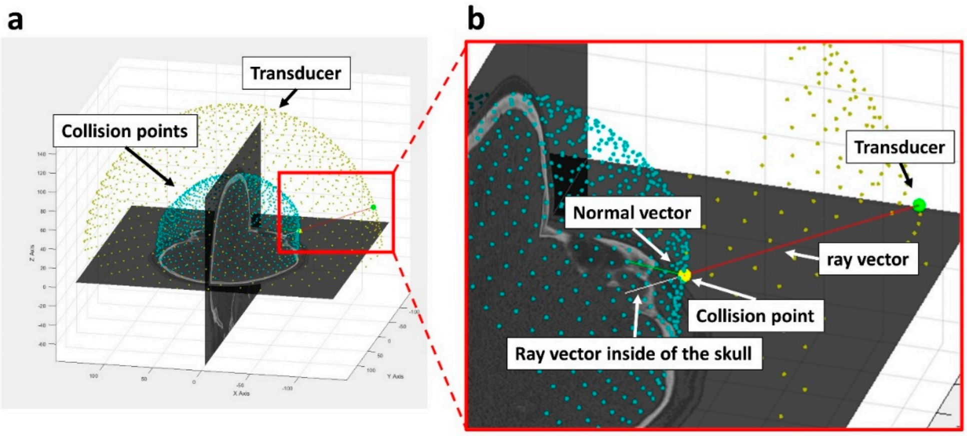

Figure S2. Normal vector based on the collision point. The normal vector of the skull surface can be derived by applying the 3D edge operator to the defined collision point. a) Illustration of derived normal vector with CT slice images. b) Zoom-in of (a) for the clear view of derived normal vector.

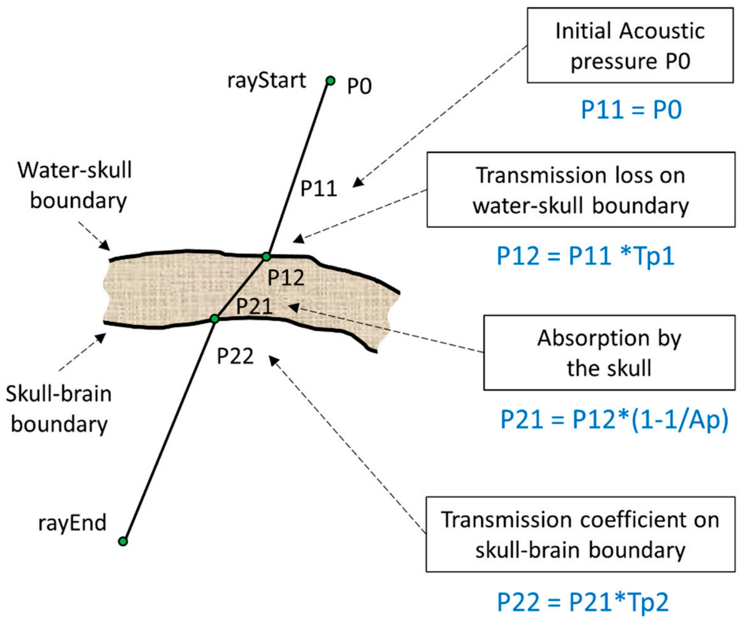

Figure S3. An illustration of total transmission coefficient calculation strategy. Tp1 and Tp2 indicate the transmission coefficients through outer and inner skull layers, respectively. The absorption coefficient caused by the heterogeneous skull structure was defined as Ap.

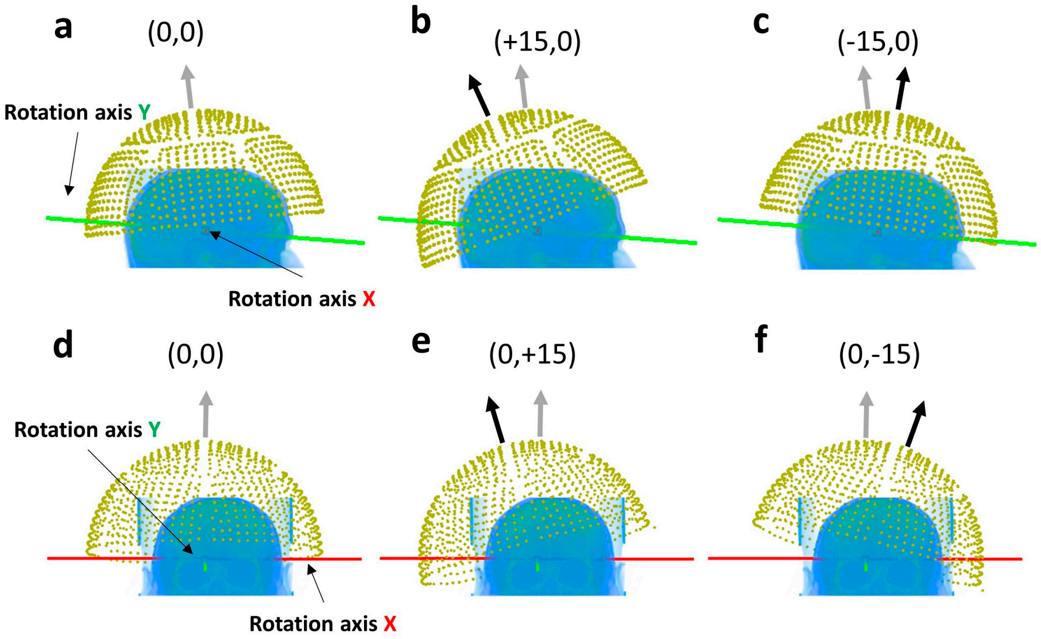

Figure S4. The implementation of transducer tilting in KranionM. b) and c) show the transducer tilting on X axis (frontal axis) which illustrated using red line. e) and f) show the transducer tilting on Y axis (Sagittal axis) which illustrated using green line.
